# Molecular and clinical analysis of Ellis-van Creveld syndrome in the United Arab Emirates

**DOI:** 10.1186/1471-2350-11-33

**Published:** 2010-02-25

**Authors:** Bassam R Ali, Nadia A Akawi, Faris Chedid, Mahmood Bakir, Moghis Ur Rehman, Aiman Rahmani, Lihadh Al-Gazali

**Affiliations:** 1 Department of Pathology, Faculty of Medicine and Health Sciences, United Arab Emirates University, PO Box 17666, Al-Ain, United Arab Emirates; 2 Department of Paediatrics, Tawam Hospital, Al-Ain, United Arab Emirates; 3 Department of Paediatrics, Al-Ain Hospital, Al-Ain, United Arab Emirates; 4 Department of Paediatrics, Faculty of Medicine and Health Sciences, United Arab Emirates University, PO Box 17666, Al-Ain, United Arab Emirates

## Abstract

**Background:**

Ellis-van Creveld (EvC) syndrome is an autosomal recessive chondrodysplastic condition with clinical manifestations that include short-limbs and ribs, postaxial polydactyly and dysplastic nails and teeth. In about two thirds of patients, mutations in either *EVC *or *EVC2 *genes have been found to be the underlying cause.

**Methods:**

In this paper, we describe the molecular (DNA sequencing) and clinical analysis of six children diagnosed with EvC from four different families from the United Arab Emirates (UAE).

**Results:**

All the children had the common clinical and radiological features of this syndrome. However, DNA sequence analysis of the genes shown to be involved (*EVC *and *EVC2*) revealed a novel splice site mutation (c.2047-1G>T) in intron 13 of *EVC2 *gene in one family. In addition, we confirm previous mutational analyses that showed a truncating mutation in exon 13 of *EVC *gene (c.1813C>T; p.Q605X) in the second family and a single nucleotide deletion (c.981delG; p.K327*fs*) in exon 8 of *EVC2 *gene in the third family. No mutations in the exons, splice sites or the promoter regions of either gene have been found in the index case of the fourth family who exhibited "EvC-like" features.

**Conclusions:**

Given the small population size of UAE, our data illustrates further the molecular heterogeneity observed in EvC patients and excludes the possibility of a common founder effect for this condition in the UAE reflecting the current ethnic diversity of the country.

## Background

Ellis-van Creveld syndrome (EvC, MIM 225500) is a chrondro-ectodermal dysplasia characterized by Richard Ellis and Simon van Creveld [1]. This condition is inherited in an autosomal recessive manner and typically involves malformations of certain cartilages especially those derived from "neural crest" cells, polydactyly and congenital heart defects in about 50 to 60% of the affected individuals [2]. EvC is a relatively rare disorder with only around 300 cases reported worldwide and is most prevalent in the Amish population of USA and some Arab populations [3-5]. Consanguinity has been reported in about 30% of the cases [6]. The birth prevalence in non-Amish population is estimated to be 0.7/100,000 of live birth [7]. However, in the UAE, Al-Gazali *et al*. [4] calculated the birth rate of this syndrome to be 5.2/100,000 of live births, representing a useful baseline for this group of birth defects in the local society.

The EvC underlying gene was mapped to chromosome 4p16 [8] and mutations have been found in the EVC gene by Ruiz-Perez et al. [3]. Mutations in a second gene EVC2, which is arranged in a head to head orientation with the *EVC gene on chromosome 4, have also been identified as causative *of the disease in a number of patients [9]. More recently, more mutations have been reported in both *EVC *and *EVC2 *in two thirds of EvC patients suggesting molecular heterogeneity of this condition and the possibility of the involvement of other gene(s) [10]. While *EVC *gene has 21 coding exons that produce a 992 amino acid protein, *EVC2 *gene has 22 coding exons and encodes a 1,308 amino acid protein without any homology to each other or to any other protein family [11]. In most cases, affected individuals with mutations in *EVC *and *EVC2 *have the typical spectrum of features and are phenotypically indistinguishable [11]. However, recent findings by Ulucan *et al*. [6] indicated that there is a considerable phenotypic variation in EvC highlighted by the analysis of a large family that exhibited complex septal cardiac defects, rhizomelic limb shortening and polydactyly without the typical lip, dental or nail abnormalities. The underlying genotype for this milder phenotype was reported to be a missense mutation (p.L623P) in *EVC *gene [6]. In this study we report the molecular analysis and clinical phenotyping of six individuals from the UAE with EvC. Our main findings are the identification of a novel splice site mutation in *EVC2 *gene and the illustration of the molecular heterogeneity of this condition among UAE patients.

## Methods

### Patients

**Figure 1:**
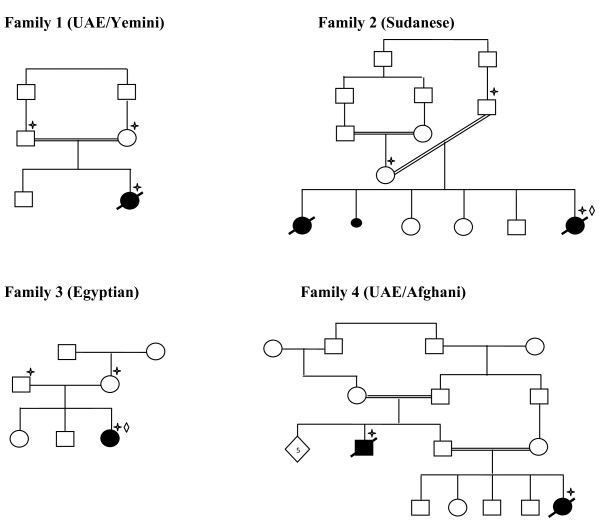
**Pedigrees of the families involved in this study**. The pedigrees shows consanguinity in three of the families affected with EvC. The plus sign indicates that the individual have been tested by the authors and the diamond indicates that the individual have been reported in Tompson et al [10].

The research was approved by Al Ain Medical District Human Research Ethics Committee (07/114 and RECA/95/19). Patients were evaluated clinically by experienced neonatologists and a clinical geneticist. The criteria for inclusion were either: 1) postaxial polydactyly and radiographic evidence of EvC (short limbs, short ribs and characteristic pelvis), or 2) short limbs and short ribs with dysplastic nails or multiple frenulae if radiographs were not available [10]. Informed consent was obtained from the parents. The pedigrees of families affected are shown in Figure 1.

### Genomic DNA Isolation

Genomic DNA was extracted from whole blood using the Flexigene DNA Kit (Qiagen, GmbH) according to the manufacturer's instructions or other standard technique.

### Polymerase Chain Reaction (PCR)

The primers shown in additional file 1 were designed using Primer3 http://frodo.wi.mit.edu/primer3/ and used to screen genomic DNA for mutations in the promoter regions and all coding exons and at least 100 bp of the flanking intron regions including the splice sites of *EVC *and *EVC2 *genes http://genome.ucsc.edu/. The promoter regions of *EVC *and *EVC2 *genes were predicted using the free online Proscan Version 1.7 software http://www-bimas.cit.nih.gov/molbio/proscan/. PCR reactions consisted of 1.5 μM MgCl_2_, 5 μM each of forward and reverse primers, 100 ng DNA template, 0.2 mM of each dNTP, 0.5 Units Taq DNA Polymerase (Qiagen, GmbH) and 1× PCR buffer (Qiagen, GmbH), in a total volume of 20 μl. Reactions were started by 10 min DNA denaturation at 95°C, followed by 40 cycles of the following PCR programme: 30 sec at 95°C, 45 sec at 58°C, and 45 sec at 72°C; the PCR was completed by a single cycle of 7 min at 72°C.

### DNA sequencing

After PCR, products were cleaned by treatment with ExoSAP-IT according to the manufacturer's protocol (USB, USA). Both strands of each amplicon were sequenced using the corresponding forward and reverse PCR primers (additional file 1). 10 μl of cycle sequencing reactions consisted of: 4 μl BigDye Mix (ABI, Foster City, CA); 1 μl primer (3.2 μM); and 1-3 μl PCR product were cycled 25 times10 sec at 96°C, 5 sec at 50°C, and 4 min at 60°C. Purified reactions were then analyzed on an ABI 3130*xl *Genetic Analyzer (ABI, Foster City, CA) based at the Faculty of Medicine and Health Sciences, UAE University.

## Results

### Patients' phenotypes

The clinical phenotypes of the patients are summarized in table 1 and detailed bellow:

**Table 1:** Clinical features of cases from 4 families with EvC syndrome.

Features	Case 1	Case 2	Case 3	Case 4
Ethnic origin	UAE/Yemini	Sudan	Egypt	UAE/Afghani

CHD	Common atrium with dilated coronary sinus	?	AV canal defect	ASD

Facial Features				

Short/thin upper lip	+	+	+	+

Short/multiple frenula	+	+	+	+

Irregular alveolar ridge	+	+	+	+

Natal teeth	-	+	+	+

Short broad nose	+	+	+	+

Long philtrum	+	+	+	+

Skeletal features				

Postaxial polydactyly	Hands, right	Hands, bilateral	Hands, bilateral	Hands and feet, bilateral

Limb shortening	Mesomelic	Mesomelic	Mesomelic	Mesomelic

Narrow chest	+	+	+	+

Nail hypoplasia	+	+	+	+/-

Radiological Features				

Short long bones	+	+	+	+

Short ribs with narrow chest	+	+	+	+

Small iliac bones with downward spike	+	+	+	

#### Family 1

**Figure 2:**
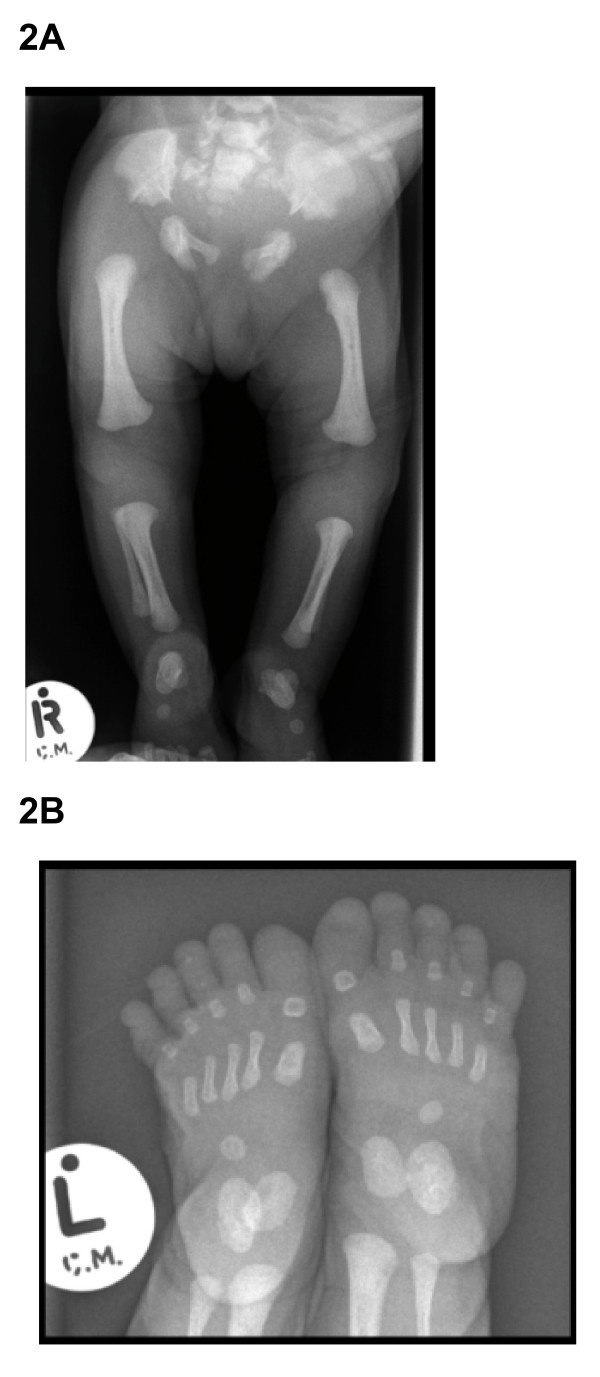
**A X-ray of the lower limbs in case 3, note small iliac bones with triradiant acetabulum, short and thick long bones**. **1B) **X-ray of the feet in case 3 showing hypoplasia of the terminal phalanges.

This baby was the second child of first cousin UAE parents of Yemini origin (Figure 1). There was no family history of similar problems and the pregnancy and delivery were normal. Multiple congenital anomalies were diagnosed prenatally. At birth the baby needed intubation and ventilation. Examination at birth revealed a weight of 2215 gm(<5%), length of 43 cm (<5%) and head circumference of 33.5 cm(<25). The baby was found to have dysmorphic features with narrow chest with short limbs (all elements), postaxial polydactyly and hypoplastic nails (Table 1). Echocardiography showed common atrium with dilated coronary sinus. The baby developed severe pulmonary hypertension secondary to lung hypoplasia which was resistant to treatment and died in the first 2 months of life. Skeletal survey showed short long bones with short ribs with narrow and long thorax. The iliac bones appeared small with a downward spike. The terminal phalanges of the toes were hypoplastic (Figure 2a &2b).

#### Family 2

The parents are first cousin once removed (Figure 1) originally from Sudan and they had 5 children, 2 of them were affected. There was a history of one miscarriage. The first affected had the typical features of EvC and died on the second day of life. No DNA was available from this child. The second affected was diagnosed prenatally by ultrasound. She died half an hour after birth. No birth measurements were available. There was distal shortening of the limbs, postaxial polydactyly of both hands with hypoplastic nails, narrow chest, and distended abdomen with enlarged liver. She had the facial and oral features of EvC syndrome (Table 1). Skeletal survey showed short long bones with short ribs with narrow and long thorax. The iliac bones appeared small with a downward spike (Table 1).

#### Family 3

This patient was the third child of unrelated parents of Egyptian origin (Figure 1). The first 2 children were healthy. There was a history of postaxial polydactyly in the paternal grandfather. The pregnancy and delivery were normal. The patient birth weight was 3600 gm (>25%), length 45 cm (<5%), and head circumference 34 cm (50%). At birth she was noted to have a narrow chest with shortening of the distal segments of all limbs with bilateral postaxial polydactyly of the hands and hypoplastic fingernails and toenails. Echocardiography showed complete AV canal defect. This was corrected at 6 months of age. Ultrasound of the brain and kidneys were normal. Skeletal survey confirmed the diagnosis of EVC syndrome (Table 1).

#### Family 4

This is a highly inbred UAE family originally from Afghanistan (Figure 1). There were 2 affected children in 2 branches (the proband and her uncle from the father's side). The proband was a female and the product of normal pregnancy and delivery. Her birth weight was 2500 gm (>5%) and length was 42 cm (<5%). She was noted to have dysmorphic features with short limbs with bilateral postaxial polydactyly of the upper and lower limbs, very narrow chest with hypoplastic lungs (Table 1). The nails were noted to be normal. Echocardiography revealed atrial septal defect (ASD). Skeletal survey showed short ribs with long and narrow thorax, short long bones and typical changes in the pelvis. She had repeated chest infections requiring intensive care unit (ICU) care on many occasions. At 2 years of age she was oxygen dependant and had normal intelligence. She died at the age of 3 years due to respiratory complications. The uncle of the proband who was the product of first cousin marriage was also affected. He was the product of normal pregnancy and delivery. At birth he was noted to have short limbs with bilateral post axial polydactyly of upper and lower limbs, hypoplastic nails, narrow chest. There were short frenula and natal teeth. There was a murmur at the left sternal edge suggestive of ASD. The baby died in the first few weeks of life due to respiratory complications. Due to lack of an obvious pathogenic mutation in either *EVC *or *EVC2 *genes we consider the diagnosis of EvC in this family tentative and therefore we refer to it as "EvC-Like" syndrome. Unfortunately, X-rays are not available for the affected members of this family.

### Patients' genotypes

**Figure 3:**
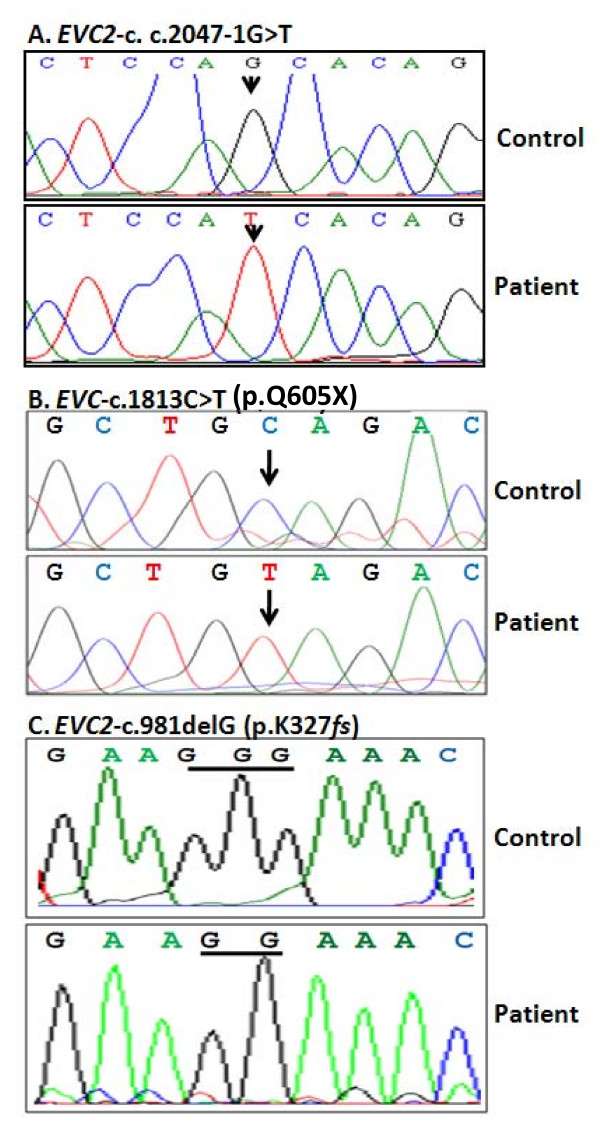
**Sequence chromatograms of mutations found in EVC or *EVC2 *genes from EvC patient from UAE**. A) Chromatograms showing the novel splice site mutation c.2047-1G>T in intron 13 of *EVC2 *gene. B) Chromatograms of the missense mutation c.1813C>T in exon 13 of *ECV *gene. C) Chromatograms of the c.981delG in exon 8 of *EVC2 *gene in case 3.

Screening for sequence variations in *EVC *and *EVC2 *genes was conducted by analyzing the DNA sequencing chromatograms for heterozygosity and alignment of the obtained sequence for each amplicon with its corresponding original genomic sequence http://genome.ucsc.edu/ using ClustalW2 http://www.ebi.ac.uk/Tools/clustalw2/index.html. We have identified a splice site homozygous change (c.2047-1G>T) in intron 13 of *EVC2 *gene for the index case of family 1 (Figure 3A). This mutation has not been found in 100 ethnically matched controls. We also confirm the presence of a missense mutation (c.1813C>T; p.Q605X) in exon 13 of *EVC *gene for the case index in family 2 (Figure 3B) and a deleted G (c.981delG; p.K327*fs*) in exon 8 of *EVC2 *gene for the index case of family 3 (Figure 3C); presence of these mutations in members of those families have been previously reported [10]. The parents were tested where DNA samples were available and found to be heterozygous for the relevant mutation (not shown). Single nucleotide polymorphisms (SNPs) in *EVC *and *EVC2 *genes were also identified in the index case with the novel mutation of family 1 in (Table 2). No mutations in either *EVC *or *EVC2 *genes were detected in the DNA from the index case of family 4.

**Table 2:** SNPs identified in *EVC and EVC2 *genes in the index case of family 1 with the novel mutation

dbSNPs	Gene	Reference Number	Minor Allele Frequency^a^	Average Heterozygosity^b^
**NM_153717.2:c.769 C>T**	*EVC*	rs6446393	C:0.04	--

**NM_153717.2:c.772 T>C**	*EVC*	rs6414624	T:0.13	0.351 +/- 0.229

**NM_153717.2:c.939+4T>C**	*EVC*	rs2286343	T:0.43	0.480 +/- 0.098

**NM_153717.2:c.969 T>C**	*EVC*	rs4688963	T:0.47	0.500 +/- 0.014

**NM_153717.2:c.1026 G>C**	*EVC*	rs4688962	G:0.37	0.461 +/- 0.135

**NM_153717.2:c.1854C>T**	*EVC*	rs11737221	--	--

**NM_153717.2:c.2305-8A>T**	*EVC*	rs1031919	T:0.48	0.441 +/- 0.162

**NM_153717.2:c.2894+18G>A**	*EVC*	rs2279250	A:0.43	0.499 +/- 0.020

NM_153717.2:c.*14G>A	*EVC*	rs2291151	A:0.01	0.496 +/- 0.045

NM_147127.3:c. 2151C>T*	*EVC2*	unknown	--	--

**NM_147127.3:c.3507C>T**	*EVC2*	rs12511039	C:0.50	0.482 +/- 0.093

^a^Available from http://www.ncbi.nlm.nih.gov/SNP/

^b^Available from http://www.genecards.org

*Silent change appeared in 4 out of 200 normal alleles.

## Discussion

Consanguineous marriages in Arab and Middle Eastern populations are very common leading to a disproportionate number of recessive disorders within these populations [12]. This situation is exacerbated by the limited opportunities for prenatal diagnosis, carrier screening and other preventative approaches. A prerequisite for any preventative approaches is the identification of the exact molecular defects underlying those disorders. In this paper, we aimed to identify and document the molecular causes underlying Ellis van Creveld syndrome in UAE population. The UAE population is ethnically heterogeneous with significant ethnic groups originating from Arabia, Persia, Baluchistan, Asia and East Africa. In addition, the majority of the current population is expatriates from the Indian subcontinent, other Middle Eastern countries and Europe. Therefore our findings might have impact on diagnosis and prevention of this condition in neighboring countries. Our data indicate the molecular heterogeneity of this condition in UAE population (Table 3). We identified four families with either typical features of the condition or have "EvC-like" symptoms. All affected children in this study satisfied the clinical diagnostic criteria for EvC syndrome [6,10]. However, the absence of mutations in either *EVC *or *EVC2 *genes in family number 4 makes the diagnosis likely but not confirmed in this family and we therefore refer to the condition in this family as "EvC-like" syndrome. Other conditions with similar manifestations, like Weyers acrofacial dysostosis and Jeune thoracic dystrophy, were also considered in the differential diagnosis. Weyers acrofacial dysostosis is an autosomal dominant disorder characterized by postaxial polydactyly, dysplastic nails, oligodontia with conical teeth and short stature caused by short limbs. This condition is allelic to EvC syndrome and is caused by heterozygous mutation in *EVC2 *gene [13]. Patients with this syndrome however, are less severely affected than EvC and they do not have narrow chest. In addition, the mode of inheritance is autosomal dominant rather than recessive. Jeune thoracic dystrophy is an autosomal recessive condition characterized by very narrow chest with variable shortening of the limbs. Postaxial polydactyly can also be a manifestation in half of the cases. The limb shortening is usually rhizomelic rather than mesomelic as in EvC syndrome and there is usually no oral manifestation, and no nail dysplasia [14]. All the children in this report including members of family 4 had oral manifestations and nail dysplasia making the diagnosis of Jeune thoracic dysplasia unlikely.

**Table 3:** Summary of mutations found in EvC patients from UAE.

Case	Gene	DNA change*	Protein Change	Reference
**Family 1**	*EVC2*	c.2047-1G>T	splicing	This study

**Family 2**	*EVC*	c.1813C>T	p.Q605X	Tompson *et al*., 2007

**Family 3**	*EVC2*	c.981delG	p.K327*fs*	Tompson *et al*., 2007

**Family 4**	-	unknown	NA	This study

*EVC and *EVC2 *mutations numbering is based on their cDNA sequences of the GenBank accession numbers NM_153717.2 and NM_147127.4 respectively with +1 as the A of the ATG initiation codon.

Molecular study on the 4 families identified a novel splice site mutation (c.2047-1G>T) in intron 13 of *EVC2 *gene in one of these families and confirmed two mutations in two other families; a nonsense mutation (c.1813C>T; p.Q605X) in exon 13 of *EVC *gene and a single nucleotide deletion (c.981delG; p.K327*fs*) in exon 8 of *EVC2 *gene [10]. RNA from the effected members of the family with the novel splice site mutation was not available for to demonstrate its altered RNA splicing effects. The lack of mutation in *EVC *and *EVC2 *genes in the 4^th ^family could indicate incorrect diagnosis or molecular heterogeneity of the condition. However, both affected children in this family had postaxial polydactyly, oral manifestations of EvC syndrome, limb shortening, congenital heart defect (ASD) typically seen in EvC syndrome and one of them had dysplastic nails making the diagnosis of EvC syndrome most likely and hence we refer to it to "EvC-like" syndrome. However, it is possible that lack of mutation in *EVC *and *EVC2 *genes in this family is due to genetic heterogeneity of this disorder as was suggested by Tompson *et al*. [10] who sequenced *EVC *and *EVC2 *genes in 65 individuals affected by EvC. They reported mutations in either *EVC *or *EVC2 *in only two thirds of their patients with the remaining of unknown molecular cause. From these studies, we anticipate the existence of another EvC causative gene(s). Further studies are needed to clarify the underlying cause of EvC in this group of patients.

## Conclusions

We conclude that EvC in the UAE population is heterogeneous at the molecular level which reflects the ethnic diversity of the current population in the country.

## Competing interests

The authors declare that they have no competing interests.

## Authors' contributions

Study design: BRA and LA. Clinical evaluation of patients and sample collection: LA, FC, MB, MUR and AR. Generation and analysis of data: NAA, BRA and LA. Preparation of the manuscript: BRA, LA and NAA. All the authors read and approved the final manuscript.

## Pre-publication history

The pre-publication history for this paper can be accessed here:

http://www.biomedcentral.com/1471-2350/11/33/prepub

## Supplementary Material

Additional file 1 ***EVC *and *EVC2 *PCR primers**. A table shows the primer sequences for PCR amplification of all the exons and flanking regions of *EVC *and *EVC2 *genes. Click here for file
