# Supplementary material for: Molecular and clinical analysis of Ellis-van Creveld syndrome in the United Arab Emirates
Source: BMC Med Genet. 2010 Feb 25;11:33. doi: 10.1186/1471-2350-11-33 (PMC2845574; doi:10.1186/1471-2350-11-33)
Supplement: Additional file 1 — EVC and EVC2 PCR primers. A table shows the primer sequences for PCR amplification of all the exons and flanking regions of EVC and EVC2 genes. [file 1471-2350-11-33-S1.DOC]

**Additional file 1. *EVC* and *EVC2* PCR primers**.

| **Amplicon** | ***EVC*** | ***EVC2*** |
| --- | --- | --- |
| **Promotor** | 5’-GCAGTGTCCTGGTCCTAGTG-3’(F)  3’-GCAGACTTTCTGGCCTGTG-5’(R) | 5’-AGACACCAGCTCCCCTCTTT-3’(F)  3’-CCAGACCTAGGAGCCACCTG-5’(R) |
| **Exon1** | 5’-GAGGCGCGTCTGTCTCTG-3’ (F) 3’-CCAACCAGGCTCAAGGAGT-5’ (R) | 5’-CAGATGCGGGTGTTTGTTC-3’ (F) 3’-CCCTCCCTTCCTCATTCTTT-5’ (R) |
| **Exon 2** | 5’-GTTGACTGGCAAAAGTCACG-3’(F) 3’-CTACATGACACCCGGCTTCC-5’ (R) | 5’-GTGCATGTCCTCTCCATGTG-3’ (F) 3’-AGCAGAGAGTGAGGGAGCTG-5’ (R) |
| **Exon 3** | 5’-AAATGGGATGTGGCATTTTG-3’ (F) 3’-CCCACTTTCCTCCCAATCTC-5’ (R) | 5’-CGCATTATATAAAACGGGTGTG-3’ (F) 3’-TTTCCAACTTCTGGTATTTGTGTT-5’(R) |
| **Exon 4** | 5’-GGACGGAAACTCTGTGGTGT-3’ (F) 3’-ACCTCTCTGGGCCTCTGTTT-5’ (R) | 5’-GCAGTAATCTTTCTACCCTCTGTAAAA-3’(F) 3’-ATGATCTGGGCAAAATCCAA-5’ (R) |
| **Exon 5** | 5’-GAGGGACGTGCCAATATTCT -3’ (F) 3’-CTGGGACTCACTGCACACAG-5’ (R) | 5’-AATCTGCCCTCATGCCTTCT-3’(F) 3’-CCAGAACACATTCACCTGGA-5’ (R) |
| **Exon 6** | 5’-GGAGGGAGGGAGAAGAGAAA-3’ (F) 3’-TCCCAAATGTGTGCAGAAAA-5’ (R) | 5’-ATGTGTCTGGGGTGTGGTG-3’ (F) 3’-ACAACCTCTCTTGGCAGTGG-5’ (R) |
| **Exon 7** | 5’-TACAACAACCCCCAGAGCAT-3’ (F) 3’-TGCGATAGGGGAATTCAAGT-5’ (R) | 5’-TGACCTGTGCAGCAGTTAGG-3’ (F) 3’-GACAGGCAGGACCCACAC-5’ (R) |
| **Exon 8** | 5’-GCACCGTTTGGCTTTCTTA-3’(F) 3’-AAGGATGTCAAGCCTCAGGA-5’ (R) | 5’-TGCTTGGTCTGCTCTGACAC-3’ (F) 3’-CACAGAACTGGGGGATGAAC-5’ (R) |
| **Exon 9** | 5’-TTAATGACCCTGGTGGCTTC -3’-(F) 3’-CAGCCCTCTCACTGCACA-5’ (R) | 5’-GCTTTCTCAGCACGTGGAAT-3’ 3’- TTGGCCTTATGTCACTGTCG-5’(R) |
| **Exon 10** | 5’-AGGGTCCCCACTGAAATTCT-3’ (F) 3’-TCACGGGAACAGAGCTACAA-5’(R) | 5’-AAAGACCTTTGGTTTCTGTTGC-3’ (F) 3’-AGGAGGCAAATGGACAGATG-5’ (R) |
| **Exon 11** | 5’-ATGGTTGGAGAACCTTCTGG-3’(F) 3’- AGGTTCTCGCACACACACAG-5’ (R) | 5’-GGTGCATGTTCAGTGTGTCA-3’ (F) 3’-CAGTACAAAGGAGAGGCAGGA-5’ (R) |
| **Exon 12** | 5’-CTTGTGGAGGAGAGGCAGAG-3’ (F) 3’-GGGTGTTCAGGACGTGAGAT-5’ (R) | 5’-ACAGGCAGGGAGGTTAAAGG -3 (F) 3’-TTCTGTCATAGCAGCAGAAAACA-5’ (R) |
| **Exon 13** | 5’-CCTAGCAAGCAGGATTGTTGA-3’ (F) 3’-ATGAGGACACTGAGGCTGGA-5’ (R) | 5’-ACAGGTGAGTTTGGAGACCA -3’ (F) 3’-TGGCACTTGATGGGTATCAG-5’ (R) |
| **Exon 14** | 5’-TGTTTGGGGAGCTTGTCACT-3’ (F) 3’-TGATAGGATTCGGGTTCTGG-5’ (R) | 5’-CCCACCCCCACTTAATTTTC-3’ (F) 3’-ACCACAGGGCAGGAATCTC-5’ (R) |
| **Exon 15** | 5’-CCTTTCTCCATCCCTTTTCC-3’ (F) 3’-TGGACCCTACCCTGAACATT-5’ (R) | 5’-CCCCAGGCTTTCTCTGTGT-3’ (F) 3’-TGCTTCTGTAATCGGCCACT-5’ (R) |
| **Exon 16** | 5’-GAGGCAGGGACTGGATGG-3’ (F) 3’-CTGCCCCAAACTAAAAGCAC-5’ (R) | 5’-TGGTGATTGCCTTCTTACCC-3’ (F) 3’-GCCTGCAAATGTGTCAGCTA-5’ (R) |
| **Exon 17** | 5’-TGGAGAGCTGGTGTGTCTCA-3’ (F) 3’-CGGCACCACAGAGAGATATG-5’ (R) | 5’-AGGCTGTTTGTTCTCCTCCA-3’ (F) 3’-GGGTTGCAGCCTGTTTCATA-5’ (R) |
| **Exon 18** | 5’-CGTCCACTTTGATGGAGAGG-3’ (F) 3’-GTGGTCACAGGCTGGCTTC-5’ (R) | 5’-TAGCAAGACACCCAGGAGGT-3’ (F) 3’-CTGAGTTGGAGATGCCAGGT-5’ (R) |
| **Exon 19** | 5’-CACTCACGTGGAGAGGGATT -3’ (F) 3’-TGGCCAGTGTGGAACTAGAA-5’ (R) | 5’-TGGCTGAGATGTGTGCTTCT -3’ (F) 3’-AAAAAGATGAAGTGACGTGCTG-5’ (R) |
| **Exon 20** | 5’-CTGCAAGAAGTTGTCACTTGTC -3’ (F) 3’-GACTTTTGGCACACAGCAG-5’ (R) | 5’-CGGGAACATGTTTTCCAGAC-3’ (F) 3’-ATGGGGACCCTTGTGGAC-5’ (R) |
| **Exon 21** | 5’-GGCTGCATTTTCATTTAATCC-3’ (F) 3’-GCAGGAGGGAAGGTGGAAT-5’ (R) | 5’-ACAGTGGGATTACAGGCGTA -3’ (F) 3’-CCTTTCCTTGTCACCTCCTG-5’ (R) |
| **Exon 22** | - | 5’-CACTCCAGAGGGTTCATTGC -3’ (F) 3’-CGGGCAGGAGAAAATCATC-5’ (R) |
